# Prevalence and factors associated with postpartum depression at a primary healthcare facility in Eswatini

**DOI:** 10.4102/sajpsychiatry.v25i0.1404

**Published:** 2019-10-24

**Authors:** Lindelwa P. Dlamini, Sotah Mahanya, Sizakele D. Dlamini, Mduduzi C. Shongwe

**Affiliations:** 1 Department of Nursing Science, Faculty of Health Sciences, Eswatini Medical Christian University, Mbabane, Eswatini; 2 International Advanced Program in Nursing, Department of Nursing, National Cheng Kung University, Tainan, Taiwan; 3 Good Shepherd College of Nursing, Siteki, Eswatini; 4 Department of Psychology, Faculty of Applied Social Sciences, Eswatini Medical Christian University, Mbabane, Eswatini; 5 Department of Midwifery Science, Faculty of Health Sciences, University of Eswatini, Mbabane, Eswatini

**Keywords:** maternal mental health, perinatal depression, postnatal depression, postpartum, postpartum depression

## Abstract

**Background:**

Routine mental health screening has not been integrated into maternal and child health (MCH) services in many developing countries, including in Eswatini (formerly Swaziland). As a result, the burden of postpartum depression (PPD) is not well understood and thus PPD remains untreated in such settings.

**Aim:**

To describe the prevalence and factors associated with PPD among women seeking postnatal and child welfare services at a primary healthcare facility in Eswatini.

**Setting:**

The study was conducted at the King Sobhuza II Public Health Unit in Manzini, Eswatini.

**Methods:**

This was a cross-sectional study that used convenience sampling and the Edinburgh Postnatal Depression Scale (EPDS) to screen for depression among 114 mothers during the first 6 weeks of postpartum at the King Sobhuza II Public Health Unit, Manzini, Eswatini. Multiple logistic regression analysis was conducted to determine sociodemographic and clinical factors associated with PPD.

**Results:**

A majority of the participants were older than 24 years (52.6%) and unemployed (64.9%), whereas 47.4% screened positive for PPD (≥ 13 score). Adjusting for other covariates, those who were unemployed (odds ratio [OR] = 3.20, 95% confidence interval [CI] 1.17–8.79) and with poor social support from their partners (OR = 9.41, 95% CI: 3.52–25.14) were more likely to be depressed, while those who attended antenatal classes fewer than four times were less likely to be depressed (OR = 0.32, 95% CI 0.11–0.92).

**Conclusion:**

We found a high prevalence of PPD. There is a need to introduce routine maternal mental health screening during the postpartum period to ensure early detection and treatment of PPD.

## Introduction

Postpartum depression (PPD) is one of the most common complications of childbearing, occurring in 13% of postpartum women worldwide.^1^ This is even higher in developing countries as 15.6% and 19.8% of women experience mental health disorders during pregnancy and after childbirth, respectively.^2^ Postpartum depression is defined as a major depressive episode with peripartum onset (i.e. the most recent episode occurring during pregnancy as well as in the 4 weeks following delivery), accompanied by a depressed or sad mood, marked loss of interest in virtually all activities, significant weight loss or gain, insomnia or hypersomnia, psychomotor agitation or retardation, fatigue or loss of energy, feelings of worthlessness or guilt, diminished ability to think or concentrate, and recurrent thoughts of death.^3^ In this study, we limited the onset specifier to 6 weeks after delivery because our interest was in quantifying the prevalence of the major depressive disorder during the postpartum period, which is 6 weeks. In sub-Saharan Africa, studies have reported varying prevalence of PPD: 6.6% in Uganda, 50.8% in the Democratic Republic of Congo^4^ and 33.3% in Nigeria.^5^ In KwaZulu-Natal, South Africa, a setting closer to Eswatini (formerly Swaziland), about 35.0% experience depression postpartum.^6^ These figures show that maternal mental health disorders are common in this region, disputing assumptions in 20th century literature,^7^ which postulated that somehow women in Africa were protected from perinatal mental disorders due to traditional rituals and other cultural factors.

Research^8^ has shown that PPD victims are twice as likely to experience future episodes of depression over a 5-year period. A recent systematic review^9^ revealed that the risk factors for PPD include socio-economic disadvantage, unintended pregnancy, being younger, being unmarried, lacking intimate partner empathy and support, having hostile in-laws, experiencing intimate partner violence, having insufficient emotional and practical support and, in some settings, giving birth to a female baby, and having a history of mental health problems.

Findings on the mode of delivery and PPD are mixed: one study^10^ found that vaginal delivery was a risk factor for PPD, others^11,12^ found no direct link and yet another study^13^ found that women who delivered by caesarian section were at an increased risk of PPD. In Africa, the unique risk factors for PPD include poor infant nutritional status, low infant birth weight, shorter duration of breastfeeding, diarrhoeal diseases, poor self-rated health, respiratory illness, home delivery, reduced quality of interaction between mothers and infants,^14^ and poor linkage to HIV care.^15^ However, attaining a high education level, having a permanent job and having a kind and trustworthy intimate partner are assumed to be protective against PPD.^9^

In Eswatini, mental health issues have not been in the forefront in terms of advocacy and prioritisation in the Ministry of Health (MoH)’s programming agenda, which has resulted in limited mental health service delivery to the general population. For example, the country has only one psychiatric hospital and one psychiatrist serving the whole population of about 1.1 million. Currently, there is no national mental health policy, and mental health screening during pregnancy and postpartum have not been integrated in the delivery of maternal and child health (MCH) services. Identifying and treating PPD is necessary because if severe, it places the mother at an increased risk for suicidal and infanticidal behaviour.^16^ Additionally, affected mothers cannot function properly and, as a result, their children’s growth and development may be negatively affected.^2^

Findings from this study will help provide baseline data for the MoH and the Sexual Reproductive Health (SRH) programme in Eswatini on the burden and correlates of PPD in the country. Understanding the factors associated with PPD will provide healthcare workers and programmers with indicators to include when developing maternal mental health screening tools. When clinicians are informed about the risk factors for PPD, they will be in a better position to tailor maternal mental health interventions for mothers based on those identified factors. Currently, there is limited published research from Eswatini on mental health in general, let alone on maternal mental health, which has contributed to the lack of mental health and maternal mental health policy and programme development. The only published evidence about maternal depression from Eswatini is on antepartum depression (APD),^15^ which was found to be at 22.7%. However, it is unclear whether the magnitude of the depression persists postpartum or not. As a result, we conducted this study to describe the burden of PPD and its associated factors among postpartum women seeking postnatal and child welfare services at the King Sobhuza II Public Health Unit (KS II) in Eswatini.

## Methods

### Study design and setting

A cross-sectional study design was employed within the quantitative research approach. The study was conducted at the KS II PHU. The country’s healthcare delivery system is based on the primary healthcare strategy and is loosely organised in a four-tier system. The KS II PHU is a level II facility located in the central part of Eswatini, in Manzini. This facility provides a range of services, such as antenatal care, postnatal care, family planning, child welfare, outpatient curative care, and so forth. The KS II PHU was purposively selected because among all the level II healthcare facilities in this region, it has one of the highest number of patient visits per year in this region.^17^ The Manzini region was chosen because it has the highest number of the country’s population and resembles almost all the characteristics of the other regions, as it is host to the major industrial hub as well as the biggest city (Manzini Municipality), yet it still has remote rural areas within its boundaries.^18^ The KS II PHU is located in the busiest and urban part of the city and services residents from urban, peri-urban as well as from rural surroundings of the city.

### Study population and sample size

The study population included mothers who had come to seek postnatal or child welfare services at this facility who were between 7 days and 6 weeks of the postpartum period in the months of June–July 2017. In order to be part of this study, the women had to be Swazi, be 18 years old or older and should have given birth not less than 7 days before the time of data collection, but within 6 weeks since giving birth. Women who had given birth less than 7 days before the time of data collection were excluded from the study to avoid information bias through misclassification as it would be difficult for women to distinguish postpartum blues (which usually occur in the first 3–5 days after childbirth) from depressive symptoms.

The sample size for this study was estimated using Raosoft^®^ online sample size calculator^19^ assuming the following parameters: a margin of error of 5%, confidence level of 95%, population size of 12 500 deliveries in the year 2015 in the Manzini region^17^ and an estimated response distribution of 22.7% (based on the prevalence of antepartum depression in Eswatini);^15^ the minimum desired sample size for this study was 264. However, 118 participants were approached to participate in the study, two refused and another two did not return to the private room after receiving their clinical services, making the eventual sample size for this study 114, indicating a response rate of about 97%.

### Recruitment and data collection procedures

We employed convenience sampling to recruit the women and data were collected by the first author through face-to-face interviews using a well-structured questionnaire. After permission was granted by the nurse manager of the facility, the senior nurse introduced the first author to all the mothers in the waiting area during the health education session that is usually held before the start of work on a daily basis in this facility.

Participants were identified (i.e. those carrying babies) while queuing on the bench leading to the clinic rooms and were approached to participate in the study. If the participant expressed willingness to participate, they were invited to a private room that was assigned by the facility management to conduct the interviews.

Participants were interviewed after they had already received the care they had come for at the clinic in order not to disturb the nurses’ work routine. The interviews were conducted in SiSwati and each interview took an average of 20 min including 5 min for obtaining written informed consent prior to each interview.

### Measures

A well-structured, researcher-administered questionnaire was utilised to collect the data. The questionnaire had two sections: Section A asked participants to report their sociodemographic characteristics as well as other social and clinical factors related to PPD identified during literature review and Section B consisted of the Edinburgh Postnatal Depression Scale (EPDS) with 10 items screening for PPD, with the last one also screening for suicidal ideation.^20^ Participants responded to a 4-point Likert scale (0–3). The total score was calculated by summing up all item scores with the total possible score ranging from 0 to 30. Women with an EPDS score of 13 or more were classified as having depressive symptoms.^20^ Prior to this study, the EPDS had not been tested among postpartum women in Eswatini; however, it has been widely used and validated in diverse African settings including among antepartum women in Eswatini.^15^. A systematic review and meta-analysis of 10 African studies^21^ estimated the median coefficient alpha of the EPDS to be 0.84 (interquartile range 0.71–0.87), whereas at a cut-off score of ≥ 9 among 14 African studies, it demonstrated a pooled sensitivity of 0.94 (95% confidence interval [CI]: 0.68–0.99) and a pooled specificity of 0.77 (95% CI 0.59–0.88) with higher cut-off scores yielding greater specificity against a lower sensitivity.^21^ In this study, the EPDS reached an acceptable reliability with a Cronbach’s α of 0.81.

### Data analysis

Data were analysed using IBM Statistical Package for Social Sciences (SPSS) for Windows, version 20.^22^ For bivariate analysis, we performed Pearson’s chi-square tests to examine significant sociodemographic differences by PPD status, whereas multiple logistic regression analysis was performed using the forced entry method^23^ to build the final model. We computed adjusted odds ratios (AORs) and 95% CIs at an alpha level of 0.05. Selection of variables to be included in the final model was guided by the known clinical importance of the variables (based on literature), their contribution score in the model as well as an alpha level of < 0.25 in bivariate analyses.^24^ Model diagnostics were assessed using: (1) Hosmer and Lemeshow test, *χ*^2^ (degree of freedom, *df* = 8) = 7.25, *p* = 0.51, which is > 0.05 indicating that the model fitted the data well, (2) the pseudo-*R*^2^, (3) the *p*-value of the model *χ*^2^ (both shown in Table 2) and (3) Cook’s deviance values (all < 1) and standardised residuals (all within ± 2.58). We assessed for multicollinearity between the covariates by fitting the same model using the linear regression procedure^23^ to obtain tolerance (all < 1) and variance inflation factor (VIF) values (all < 10) indicating the absence of multicollinearity in the final model.

### Ethical considerations

Ethical clearance was obtained from the National Health Research Review Board (NHRRB) in Eswatini (Ref: MH/599C/IRB0009688/NHRRB 548/17). Participation in the study was voluntary and participants were assured that they could withdraw from the study at any time. Considering the potential distressing effects of some of the questions in the EPDS, the first author (a nurse by training) debriefed all the participants after completing the interviews by offering information about where they could seek psychological help if they felt that the questionnaire had evoked some emotional distress. There were two such cases in this study, and together with 20 others who expressed suicidal ideations, they were encouraged to seek further psychological management at the National Psychiatric Referral Hospital (NPRH), which is located about 1 km from the study site. Consent was obtained from the mothers to link them with nurses at the KS II PHU who in turn referred them to the NPRH as per local referral clinical procedures. All other participants who screened positive to the EPDS were also encouraged to go for a psychological consult at the NPRH at any day following the interview.

## Results

### Participants’ characteristics

A total of 114 postpartum women participated in this study. A majority of them were older than 24 years (*n* = 60, 52.6%), were unemployed (*n* = 74, 64.9%), were multipara (*n* = 71, 62.3%), had not planned the pregnancy for the current child (*n* = 79, 69.3%), had no history of gender-based violence (*n* = 91, 79.8%) and reported receiving good social support from their partners (*n* = 68, 59.6%). In bivariate analyses, Chi-square tests showed that depressed women were statistically significantly different from those who were not depressed by employment status (*χ*^2^ = 3.78, *df* = 1, *p* = 0.05), planned pregnancy status (*χ*^2^ = 5.15, *df* = 1, *p* = 0.02) and social support from the partner (*χ*^2^ = 25.51, *df* = 1, *p* < 0.001). There were no statistically significant differences between the two groups of women with respect to other sociodemographic and clinical factors in the other bivariate analyses (Table 1).

**TABLE 1:** Distribution of participants’ characteristics by postpartum depression status (*N* = 114).

Characteristics	Total		Depressed (*n* = 54)		Not depressed (*n* = 60)		*χ*^2^	*p*
	*n*	%	*n*	%	*n*	%		
**Maternal age (in years)**							0.29	0.59
18–24	54	47.4	27	50	27	45		
25–45	60	52.6	27	50	33	55		
**Employment status**							3.78	0.05
Employed/self-employed	40	35.1	14	25.9	26	43.3		
Unemployed	74	64.9	40	74.1	34	56.7		
**Parity**							0.02	0.89
Primipara	43	37.7	20	37	23	38.3		
Multipara	71	62.3	34	63	37	61.7		
**Antenatal care attendance**							0.63	0.43
< 4 times	38	33.3	16	29.6	22	36.7		
≥ 4 times	76	66.7	38	70.4	38	63.3		
**Method of delivery**							-	0.31^a^
Vaginal delivery	104	91.2	51	94.4	53	88.3		
Caesarean section	10	8.8	3	5.6	7	11.7		
**Gestational age at delivery**							0.06	0.82
Term	94	82.5	45	83.3	49	81.7		
Pre-/post-term	20	17.5	9	16.7	11	18.3		
**Had planned the pregnancy**							5.15	0.02
Yes	35	30.7	11	20.4	24	40		
No	79	69.3	43	79.6	36	60		
**Maternal HIV status**							1.04	0.31
Positive	43	37.7	23	42.6	20	33.3		
Negative	71	62.3	31	57.4	40	66.7		
**History of GBV**							3.68	0.06
Yes	23	20.2	15	27.8	8	13.3		
No	91	79.8	39	72.2	52	86.7		
**Social support from partner**							25.51	< 0.001
Good	68	59.6	19	35.2	49	81.7		
Poor	46	40.4	35	64.8	11	18.3		
**Sex of newborn**							2.25	0.13
Male	57	50	23	42.6	34	56.7		
Female	57	50	31	57.4	26	43.3		
**Condition of baby at birth**							-	1.00†
Normal/healthy baby	107	93.9	51	94.4	56	93.3		
Sick baby/congenital abnormality	7	6.1	3	5.6	4	6.7		
**History of mental illness (excluding epilepsy)**							NC	NC
Yes	6	5.3	4	7.4	2	3.3		
No	106	93	49	90.7	57	95		
Do not know	2	1.8	1	1.9	1	1.7		

GBV, gender-based violence; HIV, human immunodeficiency virus; NC, not computed due to few observations.

† , Fisher’s exact test.

### Prevalence of postpartum depression

As shown in Table 1, nearly half (47.4%, *n* = 54) of the participants screened positive for PPD (i.e. had a score of 13 or more on the EPDS). Worth noting is that about 22 (19%) of the 114 participants also reported to have had thoughts of harming themselves (suicidal ideation) and/or the baby (infanticidal ideation) during the postpartum period (data not shown).

### Factors associated with postpartum depression

We found that those who were unemployed (AOR = 3.20, 95% CI 1.17–8.79, *p* = 0.03) versus employed, and those who reported poor social support from their partners (AOR = 9.41, 95% CI 3.52–25.14, *p* < 0.001) compared to those who had good social support, had higher odds of screening positive for PPD adjusting for other covariates in the model. To the contrary, those who attended antenatal classes fewer than four times were less likely to be depressed (AOR = 0.33, 95% CI: 0.11–0.92, *p* = 0.04) compared to those who attended antenatal classes four or more times adjusting for other covariates in the model. Other sociodemographic and clinical variables in the model did not show statistical significance (Table 2).

**TABLE 2:** Multiple logistic regression model for factors associated with postpartum depression (*N* = 114).

Variable	*β*	SE	AOR	95% CI	*p*-
**Employment status**					
Employed	-	-	Ref	-	-
Unemployed	1.12*	0.52	3.20	1.17–8.79	0.02
**Pregnancy planned**					
Yes	-	-	Ref	-	-
No	0.66	0.51	1.96	0.72–5.33	0.19
**Social support from partner**					
Good	-	-	Ref	-	-
Poor	2.24*	0.50	9.41	3.52–25.14	< 0.001
**History of GBV**					
Yes	0.89	0.59	2.44	0.76–7.81	0.13
No	-	-	Ref	-	-
**Antenatal care attendance**					
< 4 times	−1.13*	0.54	0.32	0.11–0.92	0.04
≥ 4 times	-	-	Ref	-	-

Note: Other variables were excluded based on their clinical importance, contribution score in the model and *p* > 0.25 in bivariate analysis.

AOR, adjusted odds ratio; CI, confidence interval; GBV, gender-based violence; ref, reference category; SE, standard error.

* , *p* < 0.05; *β* for constant = −2.06; *R*^2^ = 0.29 (Cox & Snell), 0.39 (Nagelkerke); Model *χ*^2^ (5) = 38.81, *p* < 0.001.

## Discussion

This study found that the prevalence of PPD is high and that PPD is associated with employment status, antenatal care attendance and social support. Even though the prevalence of PPD was found to be higher than the worldwide prevalence rates of 12% – 15%,^25^ the results are comparable to those from previous studies in the region, such as in South Africa, where PPD ranges between 21% and 47%.^26^ A recent community assessment for APD in a peri-urban area in Eswatini found a prevalence of 22.7%.^15^ Expectedly, the prevalence of depression can be higher postpartum compared to antepartum because postpartum women have undergone a recent event of childbirth, which is associated with hormonal and psychological changes that predispose them to PPD.^3,27^ Differences in prevalence rates in studies from other regions can be attributed to differences in study designs, measurement of PPD, characteristics of study populations and variations in the contextual risk factors for maternal mental health disorders in different settings.^6^ Worth noting is that in this study, the proportion of mothers who expressed thoughts of harming themselves or their infants was high. Cox and colleagues^20^ stated that participants who answered ‘yes’ to item 10 (i.e. thought of harming self or the baby) of the EPDS require further psychological evaluation to ensure their safety and that of the baby. In this study, during data collection, those mothers who screened positive and those who expressed suicidal or infanticidal ideation were referred to the NPRH for further psychological management.

We found that mothers who were unemployed were more likely to be depressed consistent with other studies.^27,28,29^ The effects of unemployment on mental health have been long established including the effects of poverty on mental health.^30^ In Eswatini, about 63% of the population lives below the poverty line,^31^ and most of the women, more especially in rural areas, are housewives, meaning they rely mainly on their spouses for financial support towards buying necessities for raising their children and therefore are prone to suffer from financial strains which may manifest as depressive illness.

Similar to previous studies,^32,33,34^ in this study, mothers who reported that the pregnancy for the current baby was unplanned had higher odds of being depressed. Unplanned and unwanted pregnancy can lead to lack of acceptance of the child and poor support from the partner which may lead to depression.^35^ In line with previous research,^27,36^ we found that mothers who reported receiving poor social support from their partners had higher odds of being depressed. Shrivastava and colleagues^37^ stated that the most important risk factor for antenatal/postnatal depression is absence of family support especially support from the partner. Eswatini is a patriarchal society, where most of the roles of child-rearing are left to the mother with little involvement of the partner more especially during the first 3 months of the baby’s life. In fact, in Swazi culture, new mothers are expected to stay indoors for the first month of the baby’s life to protect the baby from ‘catching’ evil spirits (‘*kuhabula*’). During this time (a period in which she is called an ‘*umtedlane*’), the new mother is often isolated and may even be made to sleep in a separate room as she is not expected to do any house chores in order to promote involution and perineal healing. The primipara (even if she is married) is often allowed to go and stay with her biological mother for a period of about 1–3 months immediately after childbirth so that she can be mentored on how to become a mother. It is during such times that these mothers have little contact and support from their partners, yet practical and emotional support is an important factor during the postpartum period.

Mothers who reported having had fewer than four antenatal care visits had lower odds of screening positive for PPD in our sample. Based on literature,^38,39^ we had expected that women who had more antenatal care (ANC) visits (a sign of good health-seeking behaviour) would have lower odds of being depressed due to the benefits of counselling and educational sessions about transitioning to parenthood offered during ANC, which prepares mothers for postpartum adjustment. The cross-sectional nature of the study limits us to determining temporality and thus impedes our efforts accurately to interpret this finding. For example, it is difficult to infer that women who were depressed during the postpartum period had been depressed since antepartum and therefore were the ones who had higher ANC visits. In fact, that postulation is further disputed by a Ghanaian study^40^ that did not find evidence of an association between APD and antenatal care attendance. In this study, we had asked participants whether they were diagnosed with depression during ANC or not, of which a majority (*n* = 71, 62.3%) stated that they were never screened at all for APD, while only three participants (*n* = 3) stated that they were diagnosed with APD (data not shown). The lack of data on APD status on the majority of our sample further impedes the full interpretation of this finding.

The other reason for the unexpected direction of the association between ANC attendance and PPD could be that there are no formal ANC classes about transitioning to parenthood in the public healthcare system in Eswatini, yet in literature the women would be expected to benefit from those classes by being empowered on how to develop coping motherhood strategies during the postpartum period. Eswatini does not have a preconception care programme and therefore mothers rely on their past mothering experiences or socialisations by their biological mothers on how to cope with the demands of parenthood. Therefore, we cannot rule out the possibility that the negative association might be due to residual confounding from an unmeasured factor which we did not control for in our model. We say that because in Eswatini, more especially, there is little information shared with the women during ANC about transitioning to parenthood (as might be the case in developed societies). As much as there are usual morning group health education sessions during each ANC visit, those sessions are usually about general MCH topics and not specific to pregnant women as the audiences comprise all women (including non-pregnant ones) who have come for different MCH services. Other than that, it is also possible that the unexpected inverse association between ANC attendance and PPD could also be due to lack of statistical power in this study. We suggest that future studies with larger samples explore this association even further.

### Strengths and limitations

To our knowledge, this study is among the first to be published on PPD in Eswatini, which makes our findings important for the MoH programming as it provides baseline data on maternal mental health in the country. We utilised a high volume facility in the central part of Eswatini and in a region that resembles the country’s demographic distribution, which enhances generalisability of our findings within the Manzini region. That said, the nature of the study design cannot be immune from limitations. Firstly, we acknowledge that the limited sample size in this study might have reduced the study’s power to detect statistically significant differences between groups. Even though we attempted to demonstrate the robustness of our conservative sample size by fitting a stable regression model on the data, which enhanced the internal validity of our study, the small numbers and resulting large CIs call for cautious interpretation of the findings. The small sample size also limited adjustment of more confounders, which makes our study not to be free from residual confounding.

Secondly, the convenience sampling technique used in this study does not free the study from selection bias.

However, we believe that this did not impact on our findings as we report a very low rate of refusals, of which we would not expect the four participants who declined participation to have been significantly different by a confounding factor from those who remained. We say that because those who refused cited lack of time as a reason and not some other factor that was related to the outcome variable. Thirdly, this was a cross-sectional study and therefore temporality and causality could not be established.

### Recommendations

Despite the weaknesses mentioned previously, we have recommendations to make based on these study findings. The high levels of PPD and suicidal ideation among our sample call for qualitative studies to investigate the psychosocial challenges faced by postpartum women in Eswatini in order to develop psychosocial programmes that will help reduce the incidence of PPD among new mothers. Future studies with larger sample sizes should also investigate the association between antenatal care attendance and postpartum depression. Lastly, the Ministry of Health should consider integrating mental health screening into routine MCH care in order to detect and treat cases of PPD early. Such screening can also be extended to the antepartum period in order to identify mothers at risk of PPD early and institute prophylactic measures.

## Conclusion

We found a high prevalence of PPD in our sample. Unemployment, unplanned pregnancy and poor social support from partners were found to be the risk factors for PPD. However, attending antenatal care classes fewer times than the WHO-recommended minimum of four visits was found to be protective against PPD. There is a need for longitudinal studies with large samples drawn from the four regions in Eswatini that will examine the mechanisms through which the identified factors influence the development of PPD.
